# “Proof-Of-Concept” Evaluation of an Automated Sputum Smear Microscopy System for Tuberculosis Diagnosis

**DOI:** 10.1371/journal.pone.0050173

**Published:** 2012-11-29

**Authors:** James J. Lewis, Violet N. Chihota, Minty van der Meulen, P. Bernard Fourie, Katherine L. Fielding, Alison D. Grant, Susan E. Dorman, Gavin J. Churchyard

**Affiliations:** 1 London School of Hygiene and Tropical Medicine, London, United Kingdom; 2 Aurum Institute, Johannesburg, South Africa; 3 University of Pretoria, Pretoria, South Africa; 4 Johns Hopkins University, Baltimore, Maryland, United States of America; 5 School of Public Health, University of Witwatersrand, Johannesburg, South Africa; San Francisco General Hospital, University of California San Francisco, United States of America

## Abstract

**Background:**

“TBDx” is an innovative smear microscopy system that automatically loads slides onto a microscope, focuses and digitally captures images and then classifies smears as positive or negative using computerised algorithms.

**Objectives:**

To determine the diagnostic accuracy of TBDx, using culture as the gold standard, and compare this to a microscopist's diagnostic performance.

**Methods:**

This study is nested within a cross-sectional study of tuberculosis suspects from South African gold mines. All tuberculosis suspects had one sputum sample collected, which was decontaminated prior to smear microscopy, liquid culture and organism identification. All slides were auramine-stained and then read by both a research microscopist and by TBDx using fluorescence microscopes, classifying slides based on the WHO classification standard of 100 fields of view (FoV) at 400× magnification.

**Results:**

Of 981 specimens, 269 were culture positive for *Mycobacterium tuberculosis* (27.4%). TBDx had higher sensitivity than the microscopist (75.8% versus 52.8%, respectively), but markedly lower specificity (43.5% versus 98.6%, respectively). TBDx classified 520/981 smears (53.0%) as scanty positive. Hence, a proposed hybrid software/human approach that combined TBDx examination of all smears with microscopist re-examination of TBDx scanty smears was explored by replacing the “positive” result of slides with 1–9 AFB detected on TBDx with the microscopist's original reading. Compared to using the microscopist's original results for all 981 slides, this hybrid approach resulted in equivalent specificity, a slight reduction in sensitivity from 52.8% to 49.4% (difference of 3.3%; 95% confidence interval: 0.2%, 6.5%), and a reduction in the number of slides to be read by the microscopist by 47.0%.

**Discussion:**

Compared to a research microscopist, the hybrid software/human approach had similar specificity and positive predictive value, but sensitivity requires further improvement. Automated microscopy has the potential to substantially reduce the number of slides read by microscopists.

## Introduction

**Figure 1:**
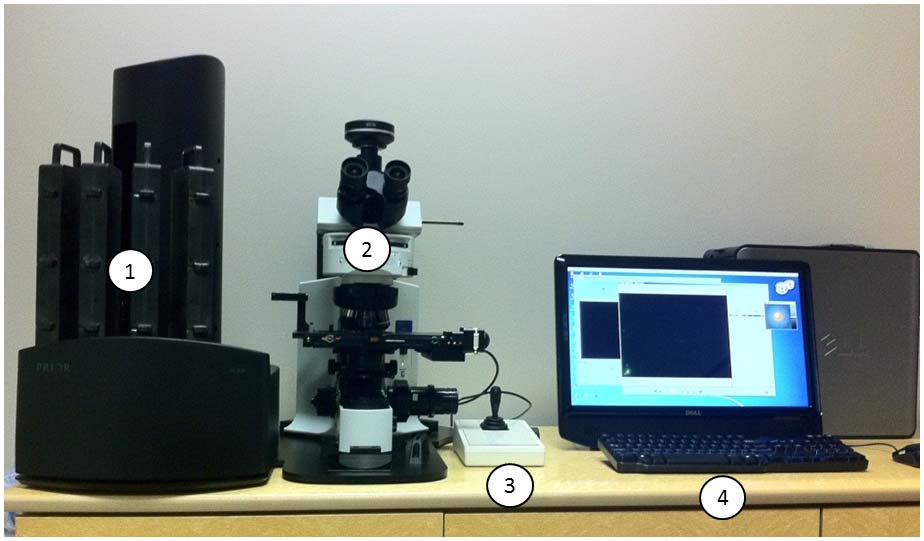
The TBDx system, including microscope, slide rack and computer system. Label 1: Prior 200 Slide Loader, with 4 slide cassettes containing 50 slides each. Label 2: Olympus Microscope, Olympus Camera and Prior Automated Slide Stage. Label 3: Joystick for manual stage movement. Label 4: Computer running TBDx integration, detection, and reporting software.

Despite early indications of a global reduction in tuberculosis incidence, in 2010 there were still an estimated 8.8 million incident cases and 1.5 million deaths worldwide [1]. Smear microscopy remains the mainstay of tuberculosis diagnosis in most high-burden settings, with 2.6 million new sputum smear-positive cases of pulmonary tuberculosis reported to the World Health Organisation in 2010 [1]. However, the sensitivity of smear microscopy is highly variable [2] for a variety of reasons, including poorly trained staff working long hours with a near absence of quality assurance [3], [4]. Mycobacterial culture remains the gold standard for diagnosing TB; however, liquid culture is associated with high levels of contamination, solid culture is slower and less sensitive, and in general the use of culture is limited to specialised laboratories with appropriate biosafety infrastructure [5], [6]. Although new technologies, such as the Xpert MTB/RIF test [7], are becoming available it is unlikely that these technologies will be affordable replacements for smear microscopy in many high burden settings for the foreseeable future and some may not be suitable for treatment monitoring [8]. “TBDx” is a new smear microscopy system that automatically: loads slides onto the stage of a conventional fluorescence microscope; focuses; digitally captures images; and then uses computerised algorithms to count the number of acid-fast bacilli (AFBs) detected to classify smears as positive or negative (Figure 1).

The aim of this study was to describe the performance characteristics and microscopist's workload of a diagnostic algorithm using TBDx alone, or in combination with a microscopist, using mycobacterial culture results as the gold standard.

## Methods

### Ethics statement

This study was nested within a cross-sectional study to evaluate the Hain MTBDR*plus* diagnostic test (as detailed below). Informed consent was obtained from all participants for this original evaluation and this consent allowed for use of the collected sputum for the performance of multiple, unspecified, tuberculosis diagnostic tests for research purposes. This consent was obtained as written consent or, for illiterate participants, witnessed oral consent. For illiterate participants, there was an impartial witness present during the consenting process, who then signed the relevant witness section of the consent form. Ethics approval was obtained for this secondary use of the study data from the same ethics committees that approved the original evaluation, namely: the University of KwaZulu Natal, South Africa; London School of Hygiene and Tropical Medicine, UK; and Johns Hopkins University School of Medicine, USA. All ethics committees approved the consent form, including the section on the use of witnessed oral consent for illiterate participants, at the beginning of the original evaluation.

### Study population

A cross-sectional study of tuberculosis suspects was nested within a large, cluster randomised trial of community-wide isoniazid preventive therapy in the South African gold mines (the “Thibela TB” study [9]), and conducted between November 2008 and January 2010. This study compared the Hain MTBDR*plus* diagnostic test to liquid mycobacterial culture and anti MPB64 antigen-based organism identification [10]. As described elsewhere, participants were a consecutive sample of adult tuberculosis suspects identified by clinical and/or radiological findings, either through self-presentation at mine health services, during routine annual chest X-ray screening, or during screening for active tuberculosis prior to isoniazid preventive therapy; those already on tuberculosis treatment were excluded [9], [10]. Each participant had one spontaneously expectorated sputum specimen collected at the time of interview. All slides were examined by fluorescence microscopy and then stored. This set of stored slides with microscopy and culture results provided an opportunity to rapidly evaluate TBDx.

### Sample selection

For the TBDx assessment, existing sample information and slides were selected from participants in the original study [10], based on the following inclusion criteria: the culture result must have been either positive for *Mycobacterium tuberculosis* or negative (i.e. specimens were excluded if the culture was contaminated or only positive for mycobacteria other than *M. tuberculosis* complex). From those who satisfied these criteria, one thousand specimens were randomly selected, using stratified random sampling, such that the same percentage were culture positive for *M. tuberculosis* as in the wider sample.

### Conventional microbiology methods

As described previously [10], expectorated sputum specimens were digested and decontaminated using N-acetyl-L-cysteine-NaOH, centrifuged, and the pellet was suspended in approximately 1.5 ml of phosphate buffer. For each specimen, a portion of the sediment was smeared over an area approximately 2×1 cm on a glass slide. Smears were completely air-dried in a biological safety cabinet and then fixed for 2 hours on an electric slide warmer. Each slide was then stained using auramine O, decolourised with acid-alcohol, and counterstained with potassium permanganate. Microscopy was performed within 24 hours by a single study-dedicated microscopist using an Olympus BHT 100 WATT Mercury Vapour Burner microscope, fitted with an Olympus Plan 40× objective (40× magnification; 0.65 numerical aperture; 22 fieldnumber) and an Olympus WHN10X/22 eyepiece (10× magnification; 22 fieldnumber), giving 400× visual magnification. Smears were examined using systematic sweeps, and a minimum of 100 fields were examined before a smear was reported as having no AFB observed; the time to read negative smears was approximately 2–3 minutes per negative smear. Smear results were quantitated as per WHO recommendations [11]. The microscopist's qualifications included approximately 45 years in a high volume clinical mycobacteriology laboratory. A 0.5 ml portion of the sediment was inoculated in the BACTEC MGIT 960 system (BD Diagnostic Systems, Sparks, MD) and positive cultures were confirmed as *M. tuberculosis* using an anti MPB64 monoclonal antibody assay (Capilia TB, TAUNS Laboratories, Numazu, Japan).

The research microscopist's original results were used in this study. In order to ensure optimal staining before being read by the TBDx system, all stored slides were re-stained using the method described above. A quality check was conducted to ensure that smear positive slides were not adversely affected by storage or re-staining. Hence, all slides initially classified as smear positive were re-read by the same research microscopist; all but a few initially scanty slides were confirmed to be positive.

### Automated microscopy methods

The automated TBDx system (Signature Mapping Medical Sciences, Inc., a wholly owned subsidiary of Applied Visual Sciences, Inc., Herndon, Virginia, USA) is based around an Olympus BX41 microscope with a 10× eyepiece and a 40× objective lens, fitted with an Olympus XC10 colour camera to acquire the images. The 40× objective lens used was an Olympus UIS2 40× objective lens with the following specifications: UPLANFL-N (Plan, Semi Apochromat), 0.75 Numerical Aperture, 26.5 Field Number, 0.51 Working Distance and infinity corrected optical system. The XC10 camera specifications were: 1.4 Megapixel with 2/3 inches sensor size; Peltier Cooled CCD (10°C at 25°C ambient); resolution of 1376×1032; bit depth used at acquisition was 12 bit; and the exposure time is approximately hundreds of milliseconds fast per field of view. The camera is mounted via an Olympus U-CMAD3 C mount adapter connected to an Olympus U-TV1x Direct Image Camera Port which is mounted onto an Olympus U-TR30 Trinocular tube, giving optical magnification of 40×. This implies that the imaged sample area is approximately 0.222×0.173 mm, or 0.038 mm^2^ per field of view. The darkfield setup utilized a 100 W Mercury Lamp housing using OSRAM bulb and an Olympus U-MWB2 Mirror Unit (Excitation Filter: 460–490; Emission Filter: 520IF; Dichromatic mirror 500).

The slide rack and loader (Prior PL-200 system) is preloaded with 1–200 slides and then the TBDx system automatically inventories and selects each slide, inserts it into the stage of the microscope (a customized version of the Prior Optiscan ES111SL), focuses the microscope, digitises 100 fields of view at 40× magnification and downloads these data to a computer, which then uses proprietary algorithms to detect and count AFBs on the digitised fields of view. Each of the 100 fields of view is analysed by TBDx, regardless of the number of AFBs detected on previously examined fields of view for that smear. Slides with no AFBs detected by TBDx are then classified as TBDx negative, 1–9 AFBs as TBDx scanty positive and ≥1 AFB as TBDx positive. TBDx reading was done blinded to all other results.

Initial piloting using a subset of slides from an earlier diagnostic sub-study within the Thibela TB study [6], read by the same microscopist, suggested that the TBDx system over-reported scanty positive smears. The TBDx system was therefore configured to present a human microscopist with digitised images from TBDx scanty slides of each field of view classified by the system to contain at least one AFB. The microscopist was then asked to confirm if each potential “AFB” on the digitised image represented true AFB or not.

### Statistical methods

Two analyses comparing diagnostic performances of the TBDx system to the microscopist were pre-specified: the first using the original reading from TBDx; and the second using the original reading from TBDx, with TBDx scanty slides reclassified as negative if the microscopist read all digitised images of potential AFBs as negative. Both analyses classified slides as TBDx positive if the final result showed at least one AFB detected in 100 fields of view.

The culture results were used as the reference for all calculations of diagnostic performance, with the denominator for sensitivity calculations being the number of specimens for which the culture was positive for *M. tuberculosis* and the denominator for specificity calculations being the number of specimens for which the culture was negative. McNemar's test was used for comparisons of sensitivity and specificity between different diagnostic methods. Fisher's exact test was used for comparisons of sensitivity and specificity between different participant characteristics (i.e. smear status, HIV status and prior history of tuberculosis). The binomial exact method was used for calculation of confidence intervals.

A sample size of 1,000 specimens with 25% culture positivity would give precision, assuming a 95% confidence interval, around a 50% sensitivity estimate of ±6.2% and precision around a 98% specificity estimate of ±1.0% and so this sample size was chosen.

## Results

### Participant and laboratory characteristics

Of the 3,165 participants who gave a sputum specimen in the cross-sectional study [10], 659 (20.8%) were culture positive for *M. tuberculosis*, 305 (9.6%) were culture positive for other mycobacterium, 1,751 (55.3%) were culture negative and 450 (14.2%) had a contaminated culture. From the 2,410 eligible specimens, one thousand specimens were randomly selected for this evaluation, nineteen of which could not be included – five slides could not be found, three were unreadable by fluorescent microscopy, two were broken or cracked and nine could not be autofocused by the TBDx system. Hence, there were 981 specimens included in this evaluation from 963 participants. As would be expected for members of the South African gold mining workforce, 95.8% of participants were male, 99.9% were Black Africans, 55.9% were South African, the median age was 45 years (inter-quartile range: 38 to 51 years) and median time in the workforce was 22 years (inter-quartile range: 12 to 30 years). Prior history of tuberculosis was reported by 27.4%. HIV status was self-reported by 411 of 981 participants (41.9%), of whom, 160 (38.9%) reported being HIV positive; 77 of these 160 (48.1%) reported currently taking antiretroviral therapy.

Of the 981 specimens, 269 were culture positive for *M. tuberculosis* (27.4%). The research microscopist graded 142 of these 269 as smear positive, giving sensitivity of the research microscopist of 52.8% (95% confidence interval [CI]: 46.6%, 58.9%; Tables 1 and 2). The remaining 712 were culture negative (72.6%), of which the research microscopist graded 10 as smear positive, giving specificity of 98.6% (95% CI: 97.4%, 99.3%).

**Table 1:** Frequency and percentage distributions of microscopist's smear status by culture results in the 981 specimens.

Microscopist reading	Culture positive for *M. tuberculosis* (n = 269)	Culture negative (n = 712)
Smear negative	127 (47.2%)	702 (98.6%)
Scanty positive	8 (3.0%)	9 (1.3%)
1+ positive	33 (12.3%)	1 (0.1%)
2+ positive	32 (11.9%)	0
3+ positive	69 (25.7%)	0

**Table 2:** The sensitivity, specificity, PPV and NPV of various microscopy methods, using culture as the gold standard.

	Sensitivity	Specificity	PPV	NPV	FoV reviewed by microscopist*
**Protocol specified (1–9 AFB = TBDx scanty):**					
Microscopist	52.8%	98.6%	93.4%	84.7%	98,100
TBDx	75.8%	43.5%	33.7%	82.7%	0
TBDx, with microscopist review of digitised images from TBDx scanty smears	61.7%	72.6%	46.0%	83.4%	1,092
**Hybrid software/human approaches:**					
A: TBDx, with microscopist review of digitised images from TBDx scanty smears (3–9 AFB = TBDx scanty)	48.0%	91.4%	67.9%	82.3%	597
B: TBDx, with microscopist's original reading of smears classified as TBDx scanty					
(1–9 AFB = TBDx scanty)	49.4%	98.9%	94.3%	83.8%	52,000
(2–9 AFB = TBDx scanty)	45.0%	99.2%	95.3%	82.7%	27,500
(3–9 AFB = TBDx scanty)	42.0%	99.2%	95.0%	81.9%	14,900

PPV = positive predictive value; NPV = negative predictive value; FoV = fields of view.

* Assumed that all smears read by the microscopist had 100 FoV reviewed per slide, as data on exact numbers read for positive smears were not recorded. Exact numbers of digital images of FoV reviewed were recorded and so these are exact.

### Pre-specified TBDx analyses

The 981 slides were assessed in April 2011 by the TBDx system, which graded 375 as negative (38.2%), 520 as scanty positive (53.0%) and 86 as positive (8.8%). Sensitivity was high (75.8%, 95% CI: 70.3%, 80.8%; Table 2), but specificity was low (43.5%, 95% CI: 39.9%, 47.3%). This translated into a similar negative predictive value [NPV] as the research microscopist, but with a much lower positive predictive value [PPV] (Table 2). Sensitivity of TBDx correlated with the smear grading of the research microscopist, increasing from 58.3% among smear negative specimens (as graded by the research microscopist), to 62.5% for scanty specimens, 75.8% for 1+ specimens, 96.9% for 2+ specimens and 100% for 3+ specimens (Fisher's exact test p-value <0.001).

Those participants who self-reported as HIV infected had similar sensitivity of TBDx as those who self-reported as HIV uninfected (HIV infected: 40/49 = 81.6%; HIV uninfected: 42/61 = 68.9%; p-value = 0.186) as well as similar specificity (HIV infected: 58/111 = 52.3%; HIV uninfected: 79/190 = 41.6%; p-value = 0.093). The same pattern was seen for the microscopist's reading for both sensitivity (HIV infected: 24/49 = 49.0%; HIV uninfected: 26/61 = 42.6%; p-value = 0.566) and specificity (HIV infected: 110/111 = 99.1%; HIV uninfected: 188/190 = 99.0%; p-value >0.99). Similarly, prior history of tuberculosis did not markedly affect the sensitivity of TBDx (prior history: 52/63 = 82.5%; no prior history: 151/205 = 73.7%; p-value = 0.180) or the specificity of TBDx (prior history: 97/205 = 47.3%; no prior history: 212/506 = 41.9%; p-value = 0.210).

Among the 520 TBDx scanty smears, there were 1,092 digital images of fields of view in which TBDx detected at least one AFB (an average of 2.1 digital images per TBDx scanty smear). The research microscopist's review of these digital images led to 245 of the 520 smears (47.1%) being re-graded as negative. This resulted in a reduction in TBDx sensitivity of 14.1%, but an increase in TBDx specificity of 29.1% (Table 2).

### Optimisation of the TBDx system

**Figure 2:**
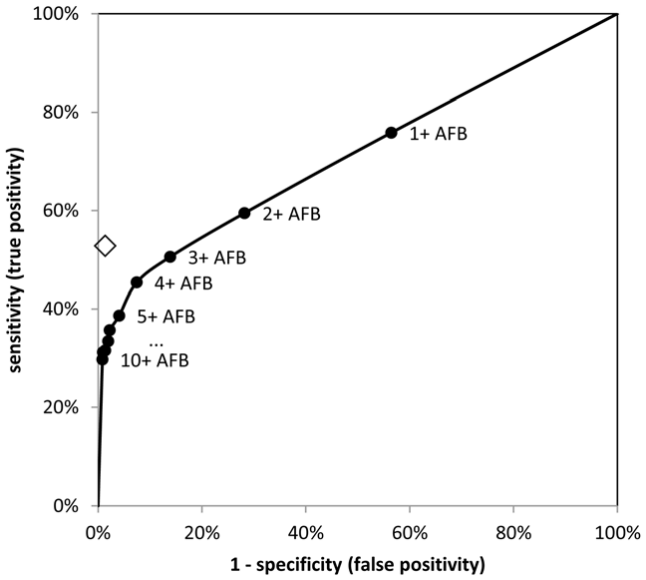
Receiver operating characteristic curve showing the influence of different cut-offs to define positivity with TBDx (circles) and contrasted to the research microscopist (diamond). AFB = acid-fast bacilli.

The results from the pre-specified TBDx algorithm gave good sensitivity, but the specificity was too low. As this was a proof of concept study, various possibilities for optimising the algorithm were subsequently investigated. Figure 2 shows the impact, on TBDx sensitivity and specificity, of increasing the number of AFBs required to define a smear as TBDx positive. To achieve TBDx specificity equal to that of the research microscopist required defining the cut-point for positive by TBDx as ≥8 AFBs per smear (i.e. smears with 0–7 AFBs classified as negative), thereby dropping sensitivity to 31.6%. To achieve TBDx sensitivity equal to that of the research microscopist, while maximising TBDx specificity, required defining the cut-point for positive by TBDx as ≥3 AFBs per smear (i.e. smears with 0–2 AFBs classified as negative). A TBDx “scanty” smear was therefore redefined as those with 3–9 AFBs.

Among the 149 smears with 3–9 AFBs detected, there were 597 digital images of fields of view in which TBDx detected at least one AFB. The research microscopist's review of these digital images led to 45 of the 149 smears (30.2%) being re-graded as negative. This hybrid software/human approach gave sensitivity of 48.0% (95% CI: 41.9%, 54.1%; Table 2 – hybrid approach A) and specificity of 91.4% (95% CI: 89.1%, 93.4%).

From a qualitative perspective, the microscopist reported finding it challenging to designate the digital images as “true AFB” or “not AFB” when visualised in isolation from the rest of the smear. Hence, three hybrid software/human approaches were explored that combined TBDx examination of all smears with the research microscopist examination of a subset of smears: smears with 0 or 0–1 or 0–2 AFBs (for the three algorithms respectively) detected by TBDx were classified as definitively negative; smears with ≥10 AFBs detected by TBDx were classified as definitively positive; and all smears with 1–9 or 2–9 or 3–9 AFBs (for the three hybrid approaches respectively) by TBDx were classified as uncertain requiring examination by the microscopist. To simulate the potential results for these hybrid approaches (hybrid approach B in Table 2), smears were not re-read by the microscopist, but instead the original microscopist reading was used. This approach gave equivalent specificity to the microscopist's readings for all three hybrid approaches. However, there was a trade-off between sensitivity and workload, with the first hybrid approach giving very similar sensitivity to the microscopist (49.4% compared to 52.8%, respectively; exact McNemar's p-value = 0.035), with almost a halving of workload from 981 to 520 smears to examine (47.0% reduction), while the third hybrid approach had lower sensitivity (42.0% compared to 52.8%, respectively; exact McNemar's p-value <0.001), but with a reduction in workload of 84.8%. These translated into very similar PPVs and NPVs between the hybrid approach and the research microscopist (Table 2).

## Discussion

This study is the first large-scale evaluation of an automated microscopy system for tuberculosis in the published literature that we are aware of. Using the fully automated TBDx system gave high sensitivity (75.8%), but low specificity (43.5%), when compared to culture as the gold standard. The low specificity was due to a large number of smears that TBDx classified as scanty positive, but were from culture negative specimens. To address this issue, a hybrid software/human approach was explored in which TBDx was used to categorise smears as strongly likely to be negative, strongly likely to be positive, or uncertain and thereby requiring examination of the smear by the microscopist. This approach (hybrid approach B) resulted in a greatly reduced workload for the microscopist while maintaining similar performance characteristics to the original microscopist's reading.

In several previously published studies of automated smear microscopy, evaluation of the performance characteristics of the automated system have used expert microscopists as the gold standard and/or used classification of individual objects as “true AFB” or “not AFB” as the primary endpoint [12]–[14]. In contrast, we have used culture results as the gold standard and we have chosen the primary endpoint to be classification of the smear as positive or negative. The use of culture as a gold standard is important as the automated microscopy system may have detected AFBs missed by the research microscopist. The use of individual objects as the primary unit of analysis can result in bias depending on how these objects were selected for evaluation. Hence, it is important to use the smear as the primary unit of analysis. In addition, a diagnosis of tuberculosis is made on the basis of positive or negative slides and not on recognition of individual AFBs, adding to the importance of evaluating the reading of smears. Both our choices of gold standard and primary endpoint represent important strengths of this study compared to previously published studies. The comparison of performance characteristics to those of a microscopist was based on the readings of a very experienced research microscopist, providing the strongest possible comparison group. The study also benefitted from a large sample size.

One potential limitation is that this study was conducted in one setting and relied on smear microscopy interpretation by one highly experienced microscopist, which may affect generalizability. However, the TBDx system was not affected by the HIV status or prior tuberculosis history of the participants from whom sputa were obtained. In many settings a “routine” microscopist might well have worse performance characteristics than the study-dedicated microscopist involved in this project; such a situation is likely to result in a better comparative performance for TBDx compared to the microscopist. In the analysis of the hybrid approach B, the use of the microscopist's original reading for slides that were classified as uncertain by TBDx has limitations. In a non-study setting, such slides may be interpreted differently by a microscopist who knows that these slides are TBDx scanty positive as the index of suspicion is likely to be higher, which may have biased our results towards great specificity and lower sensitivity. Another limitation is that the clinical data relied on self-report, including for HIV status. Finally, the choice to optimise the system for fluorescence microscopy rather than Ziehl-Neelsen may have led to reduced performance. Although sensitivity is typically higher for fluorescence microscopy [2], this is driven partly by the greater ease for the human eye to detect images using light rather than colour and this may not apply to a computerised system that can use specific colour channels. Also TBDx may have a faster processing time using bright field microscopy. Formal data on feasibility endpoints were not collected in this study. However, the current training syllabus assumes one day of training for a microscopist to use the system. Slide processing currently takes approximately two minutes (one minute for the camera to auto-focus the slide and one minute to acquire 100 digital fields of view), allowing a full slide loader of 200 slides to be processed in 6–7 hours.

A study by Somoskövi, et al. used an automated microscopy system to identify “suspected AFBs” in smears and then presented the digital images to a microscopist for review [15]. This showed very good agreement with an entirely manual reading system. A similar system was used with TBDx whereby images were reviewed by the microscopist for smears with 3–9 AFBs detected by TBDx (hybrid approach A). This gave reasonable sensitivity (48.0%) and greatly improved specificity (91.4%), with only 597 fields of view needing to be examined from 981 slides. However, the specificity was still sub-optimal and the microscopist reported difficulty in reviewing individual AFBs rather than also reviewing fields of view in other areas of the smear to have as a comparator.

If the performance of automated microscopy can equal or surpass that of an experienced microscopist, it would be useful in high volume laboratories, but potentially could also be adapted for use in smaller laboratories, for example by using the camera and processing power available in mobile phone technology to capture and analyse images without the need for more expensive computers [16]. The TBDx system is already available without the 200-slide loader and can be configured with an automated stage of one or four slide inserts, or to allow a microscopist to manually place a slide on the stage. However, although it is likely that the results presented here can be improved upon, it may not be possible to match the performance of an experienced microscopist with a fully automated system. If this is the case then one possible use of such a system would be in a hybrid software/human approach as evaluated here (hybrid approach B). This would need to have at least equal sensitivity and specificity to that of a human microscopist (using culture as the gold standard). An alternative strategy that utilises the high sensitivity, but poor specificity of fully automated TBDx, would be to reserve the use of Xpert MTB/RIF tests for specimens classified as positive on TBDx. Such a strategy could potentially greatly reduce the number of Xpert MTB/RIF tests required, making the use of the technology more affordable. With the current performance characteristics of 76% sensitivity and 42% specificity, Xpert MTB/RIF confirmation would be required for 63% of specimens. If specificity could be increased to 70% or 80% (while keeping sensitivity the same at 76%), the number of specimens requiring Xpert MTB/RIF confirmation would drop to 43% or 35%, while still detecting 76% of all culture positive cases. This would allow for the rational use of an expensive technology such as Xpert MTB/RIF. Both the combined approaches of TBDx and a microscopist and TBDx and Xpert MTB/RIF discussed here suggest potentially important roles for an automated smear microscopy system that still requires some improvement in test characteristics before it could fully replace microscopists. The internal algorithms used by TBDx to classify objects as “AFB” or “not AFB” are being refined to reduce the probability of false positives by using a stepwise classification algorithm based on a binary decision tree with feature vectors to remove different types of false positive “AFBs”, which may lead to substantial improvements in performance. The development of these internal algorithms is being done in different ways to prioritise different trade-offs of sensitivity and specificity. This will allow different configurations of TBDx depending on whether it was to be used for diagnosis (greater specificity) or screening (greater sensitivity).

Overall our findings indicate that TBDx holds promise for reducing, but probably not eliminating, the burden of manual microscopy as performed by trained microscopists. Additional work to explore colour-based staining and revised detection algorithms is underway and additional studies of TBDx deployed in field settings are warranted.

## References

[1] Stop TB Department (2011) Global tuberculosis control: WHO report 2011. Geneva: World Health Organization.

[2] Steingart KR, Henry M, Ng V, Hopewell PC, Ramsay A, et al (2006) Fluorescence versus conventional sputum smear microscopy for tuberculosis: a systematic review. Lancet Infect Dis 6: 570–581. 16931408 10.1016/S1473-3099(06)70578-3

[3] Nguyen TN, Wells CD, Binkin NJ, Pham DL, Nguyen VC (1999) The importance of quality control of sputum smear microscopy: the effect of reading errors on treatment decisions and outcomes. Int J Tuberc Lung Dis 3: 483–487. 10383060

[4] Van Deun A, Salim AH, Cooreman E, Hossain MA, Rema A, et al (2002) Optimal tuberculosis case detection by direct sputum smear microscopy: how much better is more? Int J Tuberc Lung Dis 6: 222–230. 11934140

[5] Muyoyeta M, Schaap JA, De Haas P, Mwanza W, Muvwimi MW, et al (2009) Comparison of four culture systems for Mycobacterium tuberculosis in the Zambian National Reference Laboratory. Int J Tuberc Lung Dis 13: 460–465. 19335951

[6] Chihota VN, Grant AD, Fielding K, Ndibongo B, van Zyl A, et al (2010) Liquid vs. solid culture for tuberculosis: performance and cost in a resource-constrained setting. Int J Tuberc Lung Dis 14: 1024–1031. 20626948

[7] Boehme CC, Nabeta P, Hillemann D, Nicol MP, Shenai S, et al (2010) Rapid molecular detection of tuberculosis and rifampin resistance. N Engl J Med 363: 1005–1015. 20825313 10.1056/NEJMoa0907847 PMC2947799

[8] Stop TB Department (2011) Automated real-time nucleic acid amplification technology for rapid and simultaneous detection of tuberculosis and rifampicin resistance: Xpert MTB/RIF system. Geneva: World Health Organization. 26158191

[9] Fielding KL, Grant AD, Hayes RJ, Chaisson RE, Corbett EL, et al (2011) Thibela TB: design and methods of a cluster randomised trial of the effect of community-wide isoniazid preventive therapy on tuberculosis amongst gold miners in South Africa. Contemp Clin Trials 32: 382–392. 21193066 10.1016/j.cct.2010.12.008

[10] Dorman SE, Chihota VN, Lewis JJ, van der Meulen M, Mathema B, et al (2012) Genotype MTBDRplus for Direct Detection of Mycobacterium tuberculosis and Drug Resistance in Strains from Gold Miners in South Africa. J Clin Microbiol 50: 1189–1194. 22238443 10.1128/JCM.05723-11 PMC3318561

[11] Global TB Programme, World Health Organization. Laboratory services in TB control, Part II: Microscopy. WHO/TB/98.258. Available: http://whqlibdoc.who.int/hq/1998/WHO_TB_98.258_(part2).pdf. Accessed 2012 Jan 2.

[12] Khutlang R, Krishnan S, Whitelaw A, Douglas TS (2010) Automated detection of tuberculosis in Ziehl-Neelsen-stained sputum smears using two one-class classifiers. J Microsc 237: 96–102. 20055923 10.1111/j.1365-2818.2009.03308.x PMC2825536

[13] Sadaphal P, Rao J, Comstock GW, Beg MF (2008) Image processing techniques for identifying Mycobacterium tuberculosis in Ziehl-Neelsen stains. Int J Tuberc Lung Dis 12: 579–582. 18419897 PMC4858347

[14] Veropoulos K, Learmonth G, Campbell C, Knight B, Simpson J (1999) Automated identification of tubercle bacilli in sputum. A preliminary investigation. Anal Quant Cytol Histol 21: 277–282. 10560504

[15] Somoskovi A, Gyori Z, Czoboly N, Magyar P (1999) Application of a computer-directed automated microscope in mycobacteriology. Int J Tuberc Lung Dis 3: 354–357. 10206508

[16] Breslauer DN, Maamari RN, Switz NA, Lam WA, Fletcher DA (2009) Mobile phone based clinical microscopy for global health applications. PLoS One 4: e6320. 19623251 10.1371/journal.pone.0006320 PMC2709430

